# The Emerging Jamboree of Transformative Therapies for Autoimmune Diseases

**DOI:** 10.3389/fimmu.2020.00472

**Published:** 2020-03-31

**Authors:** José M. Carballido, Camille Regairaz, Celine Rauld, Layla Raad, Damien Picard, Michael Kammüller

**Affiliations:** ^1^Translational Medicine, Novartis Institutes for Biomedical Research, Basel, Switzerland; ^2^Autoimmunity Transplantation and Inflammation, Novartis Institutes for Biomedical Research, Basel, Switzerland

**Keywords:** autoimmunity, biologics, immune checkpoints, immune tolerance, immunotherapy, nanomedicine, precision medicine, safety

## Abstract

Standard treatments for autoimmune and autoinflammatory disorders rely mainly on immunosuppression. These are predominantly symptomatic remedies that do not affect the root cause of the disease and are associated with multiple side effects. Immunotherapies are being developed during the last decades as more specific and safer alternatives to small molecules with broad immunosuppressive activity, but they still do not distinguish between disease-causing and protective cell targets and thus, they still have considerable risks of increasing susceptibility to infections and/or malignancy. Antigen-specific approaches inducing immune tolerance represent an emerging trend carrying the potential to be curative without inducing broad immunosuppression. These therapies are based on antigenic epitopes derived from the same proteins that are targeted by the autoreactive T and B cells, and which are administered to patients together with precise instructions to induce regulatory responses capable to restore homeostasis. They are not personalized medicines, and they do not need to be. They are precision therapies exquisitely targeting the disease-causing cells that drive pathology in defined patient populations. Immune tolerance approaches are truly transformative options for people suffering from autoimmune diseases.

## Introduction

Autoimmune diseases (AID) such as multiple sclerosis (MS), rheumatoid arthritis (RA), type 1 diabetes (T1D), and about 100 other conditions (https://www.aarda.org/diseaselist/), develop when certain body tissues are attacked by their own immune system. Such attack is orchestrated by autoantigen-specific T cells, generally restricted to defined major histocompatibility complex (MHC) molecules, that drive pathogenic effector T (T_eff_) cell and humoral responses (1). AID affect 5–10% of the global population and their incidence is increasing, particularly in women, who are 2–10 times more likely to develop AID than men (2). AID are chronic debilitating disorders that often associate with autoinflammatory processes and are accompanied by many comorbidities resulting in significant shortening of life expectancy. A major factor driving chronicity of these diseases and poor quality of life of the affected patients is the limited availability of transformative therapies.

Traditional treatments for AID still rely predominantly on the use of broad cytotoxic or immune suppressive chemicals (3), e.g., azathioprine, calcineurin inhibitors, corticosteroids, cyclophosphamide, leflunomide, methotrexate, mycophenolate, etc. These agents are effective in many situations and thus, they still remain a first treatment option and/or the standard of care. Recently, the Janus kinase (JAK) inhibitors have emerged as a new type of low molecular weight compounds that inhibit intracellular signal transduction of cytokine receptors. The cytokine selectivity of JAK inhibitors depends on the specificity of the different JAK family members, nonetheless, all of them inhibit the signaling of a large group of cytokines. JAK inhibitors are used for the treatment of multiple diseases (4). However, as long-term treatments, they may expose the patient to potential life-threatening opportunistic infections and risk of malignancies (4–6). Consequently, there is a clear medical need for effective, safe and specific treatments for AID. This review will discuss some of the existing and developing immunotherapy and precision medicine approaches aiming to ameliorate and/or cure AID.

## The Revolution of Biologics

About three decades ago, immunotherapy, mostly based on monoclonal antibodies (mAb), emerged as an alternative to limit the side effects associated with classical broad immune suppression (7, 8). Because of their better perceived benefit-risk profile compared to conventional therapies and their ability to control disease, the use of biologics has become widespread. This trend is expected to reinforce in the near future, due to the emergence of biosimilars and the continuous development of new biologic modalities.

Currently approved biologics target proinflammatory cytokines and mediators of inflammation and eliminate, block the functionality or the trafficking capabilities of specific leukocyte populations (Table 1). As these biologics are exquisitely selective for their targets, they are believed to have lower toxicity and better safety profiles than the classical small chemical molecules that have dominated the pharmaceutical industry in the past century. However, despite the fine specificity of these biologics for soluble molecules or cellular receptors, these treatments are not exempt of side effects. For example, as they suppress immunocompetent cell subsets, and block the action of soluble factors involved in the autoimmune attack, they also interfere with immune mechanisms that are essential in host resistance to a multitude of pathogens and thus, they increase the risk to acquire opportunistic infections, which present important complications and a potential cause of morbidity (5, 9, 10).

**Table 1:** Immunotherapy drugs approved for the treatment of autoimmune diseases^°^.

	**Target**	**Drug name**	**Biologic modality**	**First approval**	**Brand name**	**Approved indication**	**Company**
**A**							
Inhibiting cytokine signaling	BAFF/Blys	Belimumab	mAb	2011	Benlynsta	SLE	GSK
	IL-1β	Anakinra	Cytokine receptor antagonist	2001	Kineret	CAPS, RA, ScS, SD	Sobi
		Rilonacept	Cytokine receptor antagonist	2008	Arcalyst	CAPS, FCAS, Gout, MWS	Regeneron
		Canakinumab	mAb	2009	Ilaris	CAPS, FCAS, FMF, HDS, JIA, MWS, TNFR-APS	Novartis
	IL-6	Siltuximab	mAb	2014	Sylvant	MCD	Janssen (J&J)
	IL-6R	Tocilizumab	mAb	2009	Actemra	CRS, GCA, JIA, RA, SD, SS	Chugai/Genentech (Roche)
		Sarilumab	mAb	2017	Kevzara	RA	Sanofi
	IL-12/23	Ustekinumab	mAb	2009	Stelara	CD, pPso, PsA, Pso	Janssen (J&J)
	IL-17A	Secukinumab	mAb	2015	Cosentyx	AS, pPso, Pso, PsA	Novartis
		Ixekizumab	mAb	2016	Taltz	AS, pPso, PsA, Pso	Elli Lilly
	IL-17RA	Brodalumab	mAb	2017	Siliq/Kyntheum	Pso	Ortho/Leo Pharma
	IL-23	Guselkumab	mAb	2017	Tremfya	pPso, Pso,	Janssen (J&J)
		Tildrakizumab	mAb	2018	Ilumya/Ilumetri	pPso, Pso,	Sun Pharma/Almirall
		Risankizumab	mAb	2019	Skyrizi	pPso, Pso,	Abbvie
	TNF-α	Infliximab	mAb	1998	Remicade	AS, BD, BP, CD, CelD, CgD, GCA, IBD, JIA, KD, NSrc, Pcd, PF, pPso, PsA, Pso, PV, PyG, RA, SAPHO, SjS, SpA, SPD, Src, TEN, UC, Uve	Janssen (J&J)
		Infliximab biosimilars	mAb	2016	Inflectra		Pfizer
				2017	Flixabi/Renflexis		Samsung/Biogen MSD
				2017	Ixifi		Pfizer
				2018	Zessly		Sandoz (Novartis)
		Adalimumab	mAb	2002	Humira	AS, BD, CD, HS, JIA, pPso, PsA, Pso, RA, SpA, Src, UC, Uve,	Abbvie
		Adalimumab biosimilars	mAb	2014	Exemptia		Zydus Cadila
				2016	Adfrar		Torrent Pharmaceutic.
				2016	Amjevita/Solymbic		Amgen
				2016	Imraldi		Biogen
				2017	Cyltezo		Boehringer Ingelheim
				2017	Hadlima		Samsung
				2018	Mabura		Hetero
				2018	Hyrimoz		Sandoz (Novartis)
				2018	CinnoRA		CinnaGen
				2018	Hulio		Fujifilm Kyowa Kirin
				2019	Idacio		Fresenius Kabi
		Certolizumab	Pegylated Fab' Ab	2008	Cimzia	AS, CD, pPsO, PsA, RA, SpA	UCB
		Golimumab	mAb	2009	Simponi	AS, PsA, RA, SpA, UC	Janssen (J&J)
		Etanercept	Soluble receptor antagonist TNFR2_Fc	1998	Embrel	AS, AzD, BD, BP, CgS, CwP, Hct, JIA, JRA, MAS, pPso, PsA, Pso, PV, RA, SAPHO, SD, SpA, Uve	Amgem, Pfizer, Takeda
		Etanercept biosimilars	Soluble receptor antagonist TNFR2_Fc	2015	Benepali		Samsung
				2016	Erelzi		Sandoz (Novartis)
				2016	Eticovo		Samsung
**B**							
Targeting leukocyte subsets	CD20	Rituximab	mAb	1997	Rithuxan/Mabthera	CLL^*^, DLBCL^*^, FL^*^, MCL^*^, NHL^*^ BP, ES, FSG, GwP, ITP, MPA, PV, RA	Genentech (Roche)
		Rituximab biosimilars	mAb	2015	Zytus		Aryogen
				2017	Truxima/Blitima/ Ritemvia/ Rituzena		Celltrion Healthcare
				2018	Reditux		Dr Reddy's Labs
				2015	Maball		Hetero Healthcare
				2013	Mabtas		Intas Pharma
				2013	Novex		EleaPhonix
				2015	RituxiRel		Reliance
				2017	Rixathon/Riximyo		Sandoz (Novartis)
		Ocrelizumab	mAb	2017	Ocrevus	MS	Roche
		Ofatumumab	mAb	2009	Arzerra	CLL^*^	Novartis
	CD52	Alemtuzumab		2013	Lemtrada/Campath	CLL^*^ MS, RA	Sanofi
**C**							
Preventing tissue homing	CD11a	Efalizumab	mAb	2003	Raptiva	pPso, Pso (withdrawn in 2009)	Genentech (Roche) Merck Serono
	Integrin α4 chain	Natalizumab	mAb	2004	Tysabri	CD, MS	Biogen
	Integrin α4β7 chain	Vedolizumab	mAb	2014	Entyvio	CD, UC	Takeda
**D**							
Intervening with immune checkpoints	CD2	Alefacept	Soluble receptor antagonist LFA3_Fc	2003	Amevive	pPso, Pso (discontinued in 2011)	Biogen/ Astellas
	CD28	Abatacept	Soluble receptor antagonist CTLA-4_Fc	2011	Orencia	JIA, PsA, RA	BMS

*AS, Ankylosing Spondylitis; AzD, Alzheimer's Disease; BD, Behcet's Disease; BP, Bullous Pemphigoid; CAPS, Cryopyrin-Associated Periodic Syndromes; CD, Crohn's Disease; CelD, Celiac disease; CgS, Cogan's Syndrome; CRS, Cytokine Release Syndrome; ES, Evan's Syndrome; FCAS, Familial Cold Autoinflammatory Syndrome; FMF, Familial Mediterranean Fever; FSG, Focal Segmental Glomerulosclerosis; GCA, Giant Cell Arteritis; Gout, Gout; GwP, Granulomatosis with Polyangiitis; Hct, Histiocytosis; HDS, Hyperimmunoglobulin D Syndrome; IBD, Inflammatory Bowel Disease; ITP, Idiopathic Thrombocytopenic Purpura; JIA, Juvenile Idiopathic Arthritis; JRA, Juvenile Rheumatoid Arthritis; KD, Kawasaki Disease; MAS, Macrophage Activation Syndrome; MCD, Multicentric Castleman's Disease; MPA, Microscopic polyangiitis; MWS, Muckle Wells Syndrome; NSrc, Neurosarcoidosis; Pcd, Polychondritis; PF, Pulmonary fibrosis; pPso, Plaque Psoriasis; PsA, Psoriatic Arthritis; Pso, Psoriasis; PV, Pemphigus vulgaris; PyG, Pyoderma Gangrenosum; RA, Rheumatoid Arthritis; RS, Reiter's Syndrome; SAPHO, SAPHO Syndrome; ScS, Schnitzler Syndrome; SD, Still's Disease; SjS, Sjögren Syndrome; SLE, Systemic lupus erythematosus; SpA, Spondyloarthritis; SPD, Subcorneal pustular dermatosis; Src, Sarcoidosis; SS, Systemic sclerosis; TEN, Toxic epidermal necrolysis; TNFR-APS, Tumor Necrosis Factor Receptor Associated Periodic Syndrome; UC, Ulcerative Colitis; Uve, Uveitis*.

* *Cancer indications: CLL, Chronic Lymphocytic Leukemia; DLBCL, Diffuse Large B-Cell Lymphoma; FL, Follicular Lymphoma; MCL, Mantle cell Lymphoma; NHL, Non-Hodgkin's Lymphoma*.

° *Sources: www.ema.europa.eu, www.drugbank.ca, www.fda.gov, www.gabionline.net/biosimilars and web sites from pharmaceutical companies*.

In the following sections we briefly review the major mode of action (MoA) of approved immunotherapeutic biologics as well as some products currently in development.

### Inhibiting Cytokine Signaling

Tissue damage associated with AID can be mediated by a range of proinflammatory cytokines produced by cells of the innate and adaptive immune system (7, 11). Initially, a shared cytokine framework was considered to define highly conserved mechanisms of inflammation in human AID. It was thought that neutralization of one of multiple redundant nodes would suffice to disrupt the inflammatory process in a large variety of human inflammatory diseases. Clinical trials testing the efficacy of various novel cytokine or cytokine receptor inhibitors revealed however a rather different reality and pointed toward the existence of a cytokine hierarchy, which could define a disease taxonomy (7, 11). Drugs aiming to block cytokine signaling are used in a large number of AID, mostly to dampen the deleterious inflammatory milieu (Table 1A).

Amongst the first biologics targeting pro inflammatory cytokines, which have deeply influenced the management of several AID are the TNF-α blockers (7, 12). Patients with diverse AID such as psoriasis (Pso), psoriatic arthritis (PsA), RA, Crohn's disease (CD), ulcerative colitis (UC), ankylosing spondylitis (AS), juvenile idiopathic arthritis (JIA), but not MS, have benefitted from TNF-α inhibition (7). On the flip side, TNF-α blockers carry the potential to reactivate opportunistic intracellular pathogens such as *Mycobacterium tuberculosis* from dormancy leading to resurgence of tuberculosis (13, 14). Surprisingly, inhibition of IL-1 has provided limited efficacy in rheumatic diseases, but it has shown great effects in autoinflammatory conditions mediated by inflammasome activation (7, 15, 16). Antibodies targeting the IL-6 receptor have been successful in RA, but they displayed limited or no effect in other chronic inflammatory conditions (7).

Additional biologics targeting other proinflammatory cytokines (IL-12, IL-17, IL-23) have progressively emerged and are becoming the standard of care in many inflammatory conditions or AID (17, 18). Initial mAb targeting this cytokine axis, like Ustekinumab, were directed to the p40 protein, which can associate with both, p35 to form the heterodimeric cytokine IL-12, or with p19 to generate IL-23 (19). Clinical trials with Ustekinumab supported its registration for Pso, at a time that this disease was still largely considered as a T_h1_ disease. Soon thereafter, it was realized that most AID could share or be exclusively of T_h17_ origin and that targeting specifically IL-17A or IL-23 could be a more selective treatment for many of these conditions (18, 20). At that time, the most advanced immunotherapeutic in clinical trials was the IL-17A specific mAb Secukinumab, which was originally aimed to be a treatment for RA, based on the initial association of IL-17 with osteoclastogenesis (21, 22). Thus, Secukinumab was tested and proved to be highly efficacious in Pso (23). Subsequent trials with IL-17 and IL-23 specific mAbs have highlighted the relevance of IL-17A blockade and provided support demonstrating a major role for the IL-23-IL-17 axis in the pathophysiology of this disease (18). In addition, and in contrast to anti-TNF-α therapy, the composite of clinical, animal and *in vitro* data accumulated with anti-IL-17A therapy indicates a low risk for mycobacterial infection (24–26).

The examples described above illustrate how blockade of key cytokine nodes regulating the differentiation and effector responses of pathogenic cell populations can be very effective ameliorating systemic and local inflammation. However, they are only optimally efficacious in certain dermatologic and rheumatologic conditions and some diseases are still looking for the ideal treatment. For example, in the case of systemic lupus erythematosus (SLE), only an anti-B-cell activating factor (-BAFF) mAb has shown a moderate efficacy in some patients (27), whereas other indications like MS have not yet clearly benefited from targeted cytokine blockade. Anifrolumab, a human mAb to type I IFN receptor did not meet primary endpoints in an initial phase 3 trial with SLE patients. However, the drug is being reevaluated by astrazeneca.com in a subsequent study (NCT02446899) that uses different efficacy criteria.

Overall, cytokine antagonism can result in dramatic and sometimes sustained clinical responses, particularly if used at the early stages of the disease. However, such approaches may not constitute a definite cure, as they usually do not induce robust and prolonged immune regulatory mechanisms. The limitations of these therapies could be explained by the known redundancy of the cytokine pathways and/or by the differential hierarchy exerted by these cytokines in particular conditions (7, 11). Today, it is generally accepted that a better understanding of the AID endotypes will be required to select the best medication for each single patient (7, 11).

### Targeting Leukocyte Subsets

A category of biologics frequently used for the treatment of AID includes mAbs targeting specific leukocyte subsets, aiming to eliminate or inactivate these cell populations (Table 1B). This approach developed following the initial success with therapies designed for the treatment of lymphoproliferative disorders. Among the best examples of the class are the mAbs directed to the B cell receptor CD20 (i.e., Rituximab, Ocrelizumab, and Ofatumumab) that induce B cell depletion (28). Rituximab was originally developed to eliminate B-cell non-Hodgkin lymphomas (29). Subsequently, it was found to be efficacious in a series of AID through depleting autoantigen-specific B cells that i) could develop into antibody secreting cells or ii) could exert immune accessory functions such as antigen presentation or cytokine release (30, 31). Despite anti-CD20 mAb therapy causing severe B cell immunodeficiency, this approach has been validated and is currently used for the treatment of several AID such as RA, pemphigus vulgaris (PV) and MS (32).

Another registered mAb in this category is Alemtuzumab, which is an antibody directed to CD52, a receptor broadly expressed on mature leukocytes (33). Alemtuzumab was originally registered for the treatment of B-cell chronic lymphocytic leukemia under the trade name of Campath and, subsequently, relaunched as Lemtrada to treat severe cases of relapsing remitting MS (34, 35). Because of the broad expression of CD52, Alemtuzumab drives cell depletion of both B and T cells, which implies significant safety concerns after extended use.

Although not yet registered, Teplizumab is an investigational product that has been in development for many years. Teplizumab consists of a humanized, Fc receptor silenced, anti-CD3 mAb, which was derived from the mouse Orthoclone, OKT3 mAb (36). The antibody was initially intended to prevent transplant rejection, however the mouse origin and the strong ability to crosslink Fc receptors of the original product resulted in strong side effects (37). Rather than acting as a strong depleting antibody, it seems that one important MoA of Teplizumab involves the inactivation of T cells, driving them into exhausted phenotypes (38). Recently, Teplizumab has shown to delay the onset of T1D for 2 years in patients at risk of developing disease (stage 2) (39). Maintenance in these patients, mostly pediatrics, in stages that precede overt disease is considered a major improvement in the field, but it is not curative. Because the patients were only exposed to a single (14 days) treatment course, there are high expectations that repeated treatment could provide stage 2 T1D patients with a longer delay in transitioning into the overt phase of the disease (stage 3).

All drugs mentioned above, have shown efficacy in some patient populations. However, they do not distinguish pathogenic vs. beneficial cells and thus, their degree of efficacy correlates with their immunosuppressant potential.

### Preventing Tissue Homing

A way to limit general immunosuppression has been to focus the therapeutic intervention at the mechanisms controlling leukocyte migration, avoiding the recruitment of the T_eff_ cells and preventing their activation at inflammatory sites (Table 1C). One of the first biologics using this MoA was Efalizumab, which was approved in 2003, under the trade name of Raptiva, to treat Pso (40, 41). Efalizumab, binds to integrin α_L_ (CD11a), which together with CD18 constitutes lymphocyte function-associated antigen 1 (LFA-1) (42). Efalizumab blocks the binding of LFA-1 to intercellular adhesion molecule-1 (ICAM-1), thereby (i) preventing the migration of T cells into inflamed tissue and (ii) inhibiting T cell activation by antigen presenting cells. Raptiva was discontinued in 2009 based on its association with the development of progressive multifocal leukoencephalopathy (PML), a rare and usually fatal disease caused by reactivation of the human polyomavirus 2 (John Cunningham or JC virus) in the central nervous system (43, 44).

**Figure 1:**
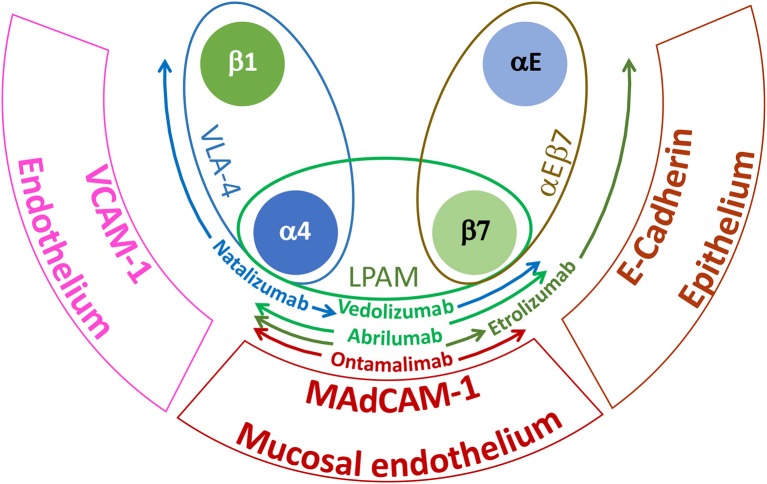
Major integrins and tissue receptors involved in leukocyte homing during IBD. VLA-4 is expressed by most leukocytes, LPAM is specifically found on lymphocytes isolated from the gastrointestinal tract and αEβ7 is displayed by intraepithelial T cells. VCAM-1 is broadly expressed by inflamed endothelium; MAdCAM-1 is selectively expressed by high endothelial venules of Peyer's patches and gut lymphoid tissues and E-cadherin is found on epithelial cells. Due to their specific target and epitope binding (see text), the indicated antibodies will be more or less selective for the gastrointestinal tissue and hence for IBD. Colored arrows indicate the spectrum of specificity for the different mAbs.

Tissue migration has also been targeted using mAbs that block the α_4_ (CD49d) and β_7_ integrin families. α_4_ integrins play a critical role in the adhesive interactions of lymphocytes with endothelial cells required for cell extravasation and migration to sites of inflammation (42). Integrin α_4_ form heterodimers with integrin β_1_ (CD29) or β_7_ to generate very late antigen-4 (VLA-4) or lymphocyte Peyer patch adhesion molecule (LPAM), respectively (42). VLA-4 interacts with endothelial vascular cell adhesion molecule 1 (VCAM-1/CD106) whereas LPAM has a preference for the endothelial receptor mucosal vascular addressin cell adhesion molecule 1 (MAdCAM-1) (42). Integrin β_7_ can also dimerize with integrin α_E_ (CD103) to form the heterodimeric receptor α_E_β_7_, which interacts specifically with E-cadherin on epithelial cells (42). There are currently two marketed mAbs directed to α_4_ integrins: Natalizumab which binds specifically to the α_4_ integrin and Vedolizumab that interacts with an epitope displayed by the heterodimer of α_4_β_7_ (45). Natalizumab was first approved for the treatment of MS (45) and subsequently, for the treatment of moderate to severe CD (46, 47). However, because prolonged therapy with Natalizumab may lead to JC virus reactivation (48–51), blocking more specifically the trafficking of leukocytes to the gut through preventing interactions with MAdCAM-1 might be a safer treatment to intervene in ulcerative colitis (UC) and CD (52). The aspiration to develop safer biotherapeutics for inflammatory bowel disease (IBD) has triggered the advancement of further additional antibodies specific for (i) α_4_β_7_ epitopes (i.e., Abrilumab by Amgen^1^) (53), (ii) integrin β_7_ (Etrolizumab, by https://www.gene.com / https://www.roche.com) (54) that will prevent interaction of α_4_β_7_ and α_E_β_7_ with MAdCAM-1 and E-cadherin, respectively or (iii) endothelial MAdCAM-1 (Ontamalimab by https://www.shire.com / https://www.takeda.com) (55) that should further increase tissue selectivity by selective blocking gut-specific integrin interaction (Figure 1).

In addition to integrins, mAbs directed to chemokines or chemokine receptors are also being considered. Many of these ligand receptor pairs have been associated with inflammatory and autoimmune processes, however their redundancy proved a real challenge for therapeutic intervention using small molecules (56). Current immunotherapy approaches aim to antagonize migratory receptors preferentially expressed by T_h1_ and T_h17_ cell subpopulations, such as CXCR3 (57–60) and CCR6 (60, 61). Most of the mAbs, currently in development, do not only block migration but mediate the killing of the target cells by antibody-dependent cellular cytotoxicity (ADCC) and/or complement-dependent cytotoxicity (CDC).

In general, these approaches have a very sound rationale. On the other hand, they suffer from the limitation that not all autoimmunity-causing T cells can be mapped using a particular integrin or chemokine receptor expression and therefore, since they still retain immunosuppressive potential, the utility of these biologics is limited to some special situations.

### Intervening With Immune Checkpoints

**Figure 2:**
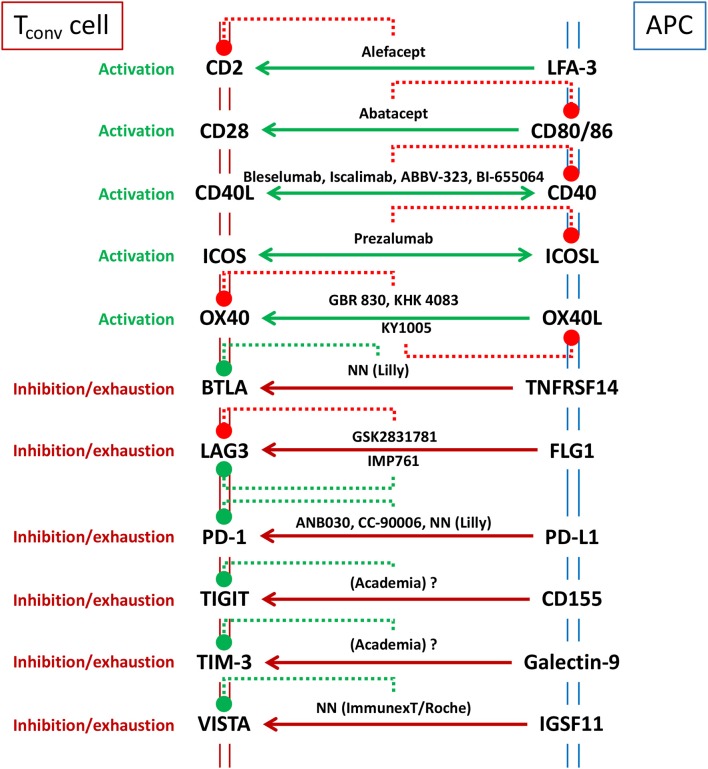
Major immune checkpoints considered as targets for immunotherapy to ameliorate AID. Solid lines represent intended targeted intervention (e.g., only targeting T cell function -unidirectional arrow to T cell side-, or also blocking helper/effector T cell function -bidirectional arrow- as in CD40/CD40L therapies that aim for the inhibition of T cell activation as well as the prevention of germinal center formation). The best known immunotherapeutics, registered or under development (see text), are included in the scheme indicating (with dotted lines) their receptor specificity and whether they are antagonist (red) or agonistic (green). GSK2831781 is displayed as a “functional” antagonist since the main function of the mAb is to eliminate LAG3 expressing cells, rather than agonizing this coinhibitory pathway, as it is the case for IMP761. NN means compound code not named (not known in public domain).

Immune checkpoints are a diverse class of receptor pairs, predominantly expressed on the surface of T cells and interacting partners, that regulate T cell activation (62, 63). Engagement of these receptors at the T cell side could be costimulatory or coinhibitory. These receptors do not act alone, they rather amplify or diminish the signals initiated by the recognition of cognate peptide-major histocompatibility complex (p-MHC) upon engagement of specific antigen-receptors on T cells in the context of a large array of cell-cell interaction bridges (63). Thus, the outcome of T cell activation does not depend on a single receptor pair but on the overall balance of a multitude of costimulatory and coinhibitory signals that occurs simultaneously during the engagement of T cells with APC or target cells. For example, costimulatory receptor bridging may dominate such balance during early phases of infection, but the equilibrium will be tilted toward coinhibitory signaling once the immune response becomes effective and pathogen loads are diminished. Such switch is designed to avoid exacerbated reactions and unnecessary host tissue damage. Unfortunately, many tumors do exploit these coinhibitory immune checkpoint pathways to elude immune surveillance. The awareness of this mechanism has sparked the use of antagonist mAbs (particularly involving cytotoxic T-lymphocyte-associated protein 4 (CTLA-4/CD152) and programmed cell death protein 1 (PD-1/CD279) or its ligand programmed death-ligand 1 (PD-L1/CD274) that prevents inhibitory receptor-counterreceptor bridging without triggering coinhibitory signals and thus, releases T cell inhibition and enhances anti-tumor immunity (62, 64). On the other hand, immunotherapeutics based on the antagonism of costimulatory immune checkpoints or on the agonism of coinhibitory counterparts would have therapeutic potential for the treatment of AID. A graphic summary of the biologics directed to immune checkpoints, already registered or under clinical development is presented in Figure 2.

#### Antagonizing Costimulatory Checkpoints

Approved examples for the antagonist of costimulation include fusion proteins consisting of extracellular domains of lymphocyte function-associated antigen 3 (LFA-3/CD58) and CTLA-4, linked to human immunoglobulin Fc domains, and are represented by Alefacept (trade name Amevive) (65) and Abatacept (trade name Orencia) (66), respectively (Table 1D).

CD2 on T cell surfaces interacts with LFA-3/CD58 during antigen presentation and thus, blockade of this costimulatory pathway using the soluble CD2 ligand, Alefacept, prevents efficient T cell activation (67). In addition, because the Fc domain of Alefacept is a wild type human IgG1, the molecule also mediates killing of memory T cells (68). Alefacept was approved for Pso (69) in the USA and a few other countries (but not in the EU market) and it was discontinued voluntarily by the manufacturer shortly after launch. Recently, Alefacept has been tested in a clinical trial for T1D. The study involved a short treatment period and showed signs of efficacy without reporting major adverse effects (70).

CD28 is a classical costimulatory molecule expressed on the surface of all naïve and most memory T cells (71). Upon T cell receptor engagement, CD28 binds to its APC counter-receptors CD80 (B7-1) and CD86 (B7-2) providing costimulatory signals required for efficient T cell activation (72). CTLA-4 is normally induced on the surface of T cells following activation. Once it is expressed, CTLA-4 also binds to CD80 and CD86 with much higher affinity than CD28, removing costimulation and providing coinhibitory signals that terminate T cell activation (73). Soluble CTLA-4_Ig (Abatacept) is a fusion molecule that uses an engineered Fc-silent domain, and thus, it inhibits CD28-mediated T cell activation without triggering cytotoxicity to target cells (74). Abatacept is an approved drug for RA (75). A drug with similar MoA, Belatacept (trade name Nulojix) has been developed specifically for the prevention of transplant rejection (76). Belatacept differs from Abatacept in two amino-acid substitutions (L104E and A29Y) (77). These modifications result in a more powerful blockade of T cell activation, especially by blocking the CD86–CD28 interaction, which in general is considered to precede CD80–CD28 due to the constitutive high abundance of CD86 on APC surfaces (78).

Interestingly, agonists of CD28 were also considered in the past for the treatment of AID, based on the observed potential to expand regulatory T (T_reg_) cells (79). However, TGN1412, a CD28 super-agonist mAb, induced a polyclonal T cell activation with a dramatic cytokine release syndrome during the first clinical trial (80, 81). TGN1412 essentially promoted T cell stimulation without simultaneous antigen-specific T cell receptor (TcR) engagement (80, 82). This unfortunate event illustrates the fact that the balance between T_reg_ and T_eff_ cell responses in laboratory animals and humans and their response to super-agonists can be significantly different (80, 83).

The CD40/CD40L costimulatory pathways have been considered since long as a potential site of intervention to prevent transplant rejection and to treat AID (84). Initial efforts focused on antagonizing CD40L (CD154), since this receptor is induced on the surface of activated T cells, which was considered an advantage over targeting the counterreceptor CD40 that is broadly expressed on APC and other cell types, i.e., endothelial cells (85). Unfortunately, clinical trials with two independent drug candidates and preclinical results with additional compounds revealed thromboembolic events, related to the expression of CD40L and FcγRII on platelets (86), and resulted in the discontinuation of these projects (87, 88). A new wave of modified antibodies and CD40L binding proteins that do not induce platelet aggregation is currently under development (84). Alternatively, there is a series of mAbs that block CD40 without causing depletion or activation of the target cells, including Bleselumab (https://www.kyowakirin.com), Iscalimab (https://www.novartis.com/), BI-655064 (https://www.boehringer-ingelheim.com in collaboration with https://www.abbvie.com), ABBV-323 (https://www.abbvie.com), ch5D12, and FFP104 (84). Particularly, Iscalimab (CFZ533) is an investigational compound that has shown to prolong the durability of transplanted kidneys and improve long-term outcomes for kidney transplant (89). Iscalimab is a fully human mAb directed to CD40. It blocks CD40-CD40L (CD154) pathway interaction without causing depletion of APC or other CD40-bearing cells. Iscalimab has shown to be effective in patients with primary Sjögren syndrome (SjS) (90) and in a subgroup of Graves' disease (GD) patients (91) and it is being explored in several other autoimmune conditions (92).

Inducible T-cell costimulator (ICOS/CD278) is another costimulatory immune checkpoint expressed on T cell surfaces, which interacts with ICOS-ligand (ICOS-L/CD275) on APC. ICOS is induced in naïve T cells upon encounter with specific antigen. Afterwards, it is expressed, and upregulated following activation, on broad subsets of antigen-experienced T cells including T_h1_, T_h2_, T_h17_, and T_reg_ cells (93). On the other hand, ICOS has been mostly implicated with follicular helper T (T_fh_) cell responses, particularly supporting survival and immunoglobulin switch of B cells in germinal centers (94–96). These characteristics drove the development and clinical investigation of Prezalumab (formerly AMG557). Prezalumab is an IgG2 mAb directed to ICOS-L that prevents productive T_fh_-B cell interactions. Prezalumab was originally tested in Pso and cutaneous lupus erythematosus by Amgen^1^. These projects were discontinued, but clinical testing resumed in collaboration with AstraZeneca^2^ for SjS, although recently AstraZeneca also halted development following poor results in a phase 2 study. AMG577 has now been re-engineered into a bispecific mAb (MEDI0700, previously AMG570) that simultaneously targets BAFF and ICOS-L (97).

Additional costimulatory immune checkpoints like OX40 (TNFRSF4/CD134), the T cell counterpart of OX40L (TNFSF4/CD252) induced during late T cell activation phases has been considered by https://www.glenmarkpharma.com/ (anti-OX40, GBR 830), https://www.kyowakirin.com/ (anti-OX40, KHK 4083) and https://www.kymab.com/ (anti-OX40L, KY1005) for the treatment of ulcerative colitis, although the emphasis of these products is to target T_h2_ mediated allergic disease.

#### Agonizing Coinhibitory Checkpoints

The alternative concept, namely agonizing immune inhibitory checkpoints, to treat AID has also been proposed in the literature in the past years (63, 98–102) and the efficacy of several coinhibitory agonists has been demonstrated preclinically *in vitro* using human peripheral blood mononuclear cells (PBMC) and *in vivo* using rodent models of AID (103–106). Among others, B- and T-lymphocyte attenuator (BTLA/CD272) (107), lymphocyte-activation gene 3 (LAG-3/CD223) (107, 108), T cell immunoglobulin and mucin-domain containing-3 (TIM-3) (107, 108), T cell immunoreceptor with Ig and ITIM domains (TIGIT) (107–114), PD1/PD-L1 (63, 101) and V-domain Ig suppressor of T cell activation (VISTA) (107, 115, 116) are emerging as very attractive target candidates for agonistic immune checkpoint intervention in AID.

BTLA has been described to inhibit T cell responses following engagement of the TNF receptor superfamily member 14 (TNFRSF14) which is also known as the herpes virus entry mediator (HVEM). A low expression of HVEM has been clinically associated to increased SLE disease activity and an elevated IFN gene signature (117). These results suggest that inefficient BTLA engagement could favor SLE and accordingly, https://www.lilly.com/ is developing an agonistic antibody to BTLA.

LAG3 is a receptor primarily found on activated T and NK cells (118) which originally was reported to interact with MHC-II molecules (119) and, more recently, has been described as a functional receptor for fibrinogen-like protein 1 (FGL1) (120). LAG3 has been implicated as a marker of exhaustion on CD8^+^ T cells (121) and as a negative regulator of T cell activation and effector function (119, 122), in addition of contributing to suppression when expressed on T_reg_ cell surfaces (123). LAG3 is being targeted in the oncology field, with compounds that aim to remove the unresponsive state of tumor specific T cells. However, it is also viewed as a candidate target for immunotherapy to treat AID. https://www.immutep.com is a biotechnology company exclusively focusing on therapeutics intervening with the LAG3 pathway. In addition of having several compounds in development for cancer, this team is working on two mAbs for AID. The most advanced compound, IMP731, is based on a T cell-depleting murine antibody, chimeric with human IgG1, that has been licensed to https://www.gsk.com (GSK2831781) and is currently in phase 2 for UC. In addition, https://www.immutep.com is developing by its own a non-depleting agonistic anti-LAG3, humanized IgG4 mAb (IMP761) for AID.

PD-1/PD-L1 is a prototype target for cancer. Antagonist mAbs to PD-1, like Pembrolizumab, Nivolumab, and Cemiplimab or to PD-L1, like Atezolizumab, Avelumab, or Durvalumab are very successful drugs treating a variety of tumors and thus, the complementary option of developing agonists counterparts for the treatment of AID has been considered by a few companies. Among those, https://www.anaptysbio.com/ has internally ANB030, and in partnership CC-90006 (https://www.celgene.com/ now part of https://www.bms.com/), agonistic anti-PD-1 projects to intervene with inflammatory diseases and Pso, respectively. https://www.lilly.com/ is also reporting the development of an agonist mAb to PD-1 for the treatment of AID.

TIGIT is a receptor absent on naïve T cells, which is induced following activation and then, remains constitutively present on subsets of memory T cells (109). TIGIT binds predominantly to the poliovirus receptor (PVR/CD155) on APCs (124). TIGIT shares this binding pattern with an additional coinhibitory receptor, T cell activation, increased late expression (TACTILE/CD96) (125), and with the costimulatory receptor DNAX accessory molecule-1 (DNAM-1/CD226) (126). CD226 is broadly expressed among all T cell subsets, although at lower intensities in the naïve populations, whereas CD96 displays also broad but low-density expression on all T cells. CD155 on the other hand is present on all APC surfaces, but it is also found in endothelial and epithelial surfaces, particularly under inflammatory conditions. The highest affinities of these receptor interactions are found between TIGIT with CD155, followed by those between CD226 with CD155, and thereafter, but weakly, by those of CD96 with CD155 (127). Furthermore, there is another inhibitory molecule on T cells, namely, poliovirus receptor related immunoglobulin domain containing (PVRIG/CD112R), which interacts with the APC counterreceptor poliovirus receptor-related 2 (PVRR2/CD112) (128), that is also a weak binder for TIGIT and perhaps also for CD226. Overall, based on receptor abundancy and affinity interactions the major players on this immune checkpoint cluster are CD155 on the APC and CD226 and TIGIT on the T cells (114). Such triad is reminiscent of the CD80/86 interactions with CD28 and CTLA-4 that tightly controls the activation of naïve (T_n_) and central memory (T_cm_) T cells. Thus, intervention with CD226/TIGIT immune checkpoints might be particularly efficient to prevent the effector functions of the CD28^−^ memory T cells (129) that are abundant in AID (130). However, there is little public information on the development of therapeutic anti-TIGIT mAbs for indications outside oncology.

TIM-3 was identified screening hybridomas from rats immunized with mouse T cells against established mouse T_h1_ and T_h2_ cell clones, followed by an expression cloning approach (131). The exercise identified TIM-3 as a molecule selectively expressed by mouse IFN-γ producing T_h1_ and T_c1_ cells but not by the T_h2_ counterparts. This pattern has been confirmed in human and extended to T_h17_ cells, that also showed TIM-3 expression although at lower abundancy (132). A series of experiments in mouse models, including the use of its ligand Galectin-9 (133) demonstrated that engagement of TIM-3 ameliorate disease by elimination of T_h1_ cells (132). In addition to Galectin-9, several ligands capable of interacting with TIM-3 have been described (100) but Galectin-9 is probably the most studied due to its broad expression in the tumor environment where it could promote inflammation or facilitate tumor escape (134). The abundancy of TIM-3 has been found to be particularly low on T cells isolated from cerebrospinal fluid of MS patients (135, 136) and from peripheral blood of RA (137) or Pso (138) subjects. Altogether, this would indicate that agonistic anti-TIM-3 mAbs might be effective eliminating disease-causing T cells. However, there are no reports in the public domain indicating drug development projects using this approach.

VISTA (also known as B7-H5) is a member of the B7 family of immunoregulatory molecules present on the surfaces of hematopoietic cells. It is expressed on myeloid cells as well as on NK cells and naïve and memory T cells but absent on B cells (139). It was originally cloned in the context of gene expression studies that compared resting vs. activated mouse CD25^+^ T_reg_ cells (115). The molecule was described to function as both, a ligand and a receptor and attributed to have immunosuppressive function based on *in vivo* studies with gene-deficient mice (139). VISTA expression was also abundant in tumor microenvironments where it facilitated tumor evasion (140) and thus antagonist anti-VISTA mAbs were considered for several malignancies. Recently, immunoglobulin superfamily member 11 (IGSF11; also known as V-set and immunoglobulin domain containing 3, VSIG3) was described to be a ligand for VISTA (141) and reported to inhibit T cell activation (142). Furthermore, antagonistic and agonistic antibodies to mouse VISTA, have been shown to enhance (143) or prevent (144) lupus, respectively, in experimental rodent models. These data support the development of agonistic anti-VISTA therapeutics for the treatment of AID. In line with these observations, http://immunext.com has an agonist anti-VISTA mAb project partnered with https://www.roche.com to intervene in AID.

Whereas, some immunotherapeutics blocking costimulatory immune checkpoints have shown efficacy in certain autoimmune conditions, the complementary intervention aiming to agonize the coinhibitory receptors seems to be lagging behind. One possible explanation could be the special challenges associated to these therapeutics that often require non-cytotoxic crosslinking of the target and necessitate to adapt their MoA depending on whether they interact with T_eff_ or T_reg_ cells.

## The Hope of Cell Therapies

The capability to counterbalance antigen-specific immune activation is critical for effective and precise immune function without inducing collateral tissue damage (145–147). T_reg_ cells expressing transcription factor forkhead box p3 (FOXP3) are a small but essential subset of lymphoid cells which are able to contract activated immune responses and maintain immune system homeostasis, thereby preventing inflammation and AID. Indeed, patients with systemic or organ-specific AID have compromised T_reg_ cell numbers and/or function (145, 146). Hence, a promising way to restrain autoimmune responses, is to expand the pools of patient's T_reg_ cells (145, 146). However, it is not always clear which subset of T_reg_ cells can or should be expanded, as an increasing diversity of immunosuppressive lymphocytes have been described (145, 146). T_reg_ cells are generally divided into two major subsets: thymus-derived cells, that normally react to self-antigens, and peripherally derived cells, that preferentially respond to “environmental” antigens (145). Peripheral CD4^+^ T_reg_ cells are classically distinguished on the basis of their constitutive CD25 (IL-2 receptor α chain) expression which parallels FOXP3 expression and immunosuppressive function (145). Another type of regulatory T cell subset, called regulatory type 1 (T_r1_) cell, does not constitutively show high expression of CD25 and FOXP3, but is characterized by surface CD49b and LAG3 and by their ability to produce abundant IL-10 and TGF-β1 with little or no IFN-γ (146, 148–150). These cells are responsible for dampening host-reactive T cells responses after hematopoietic stem cell transplantation (151–153) and for the suppression of effector autoimmune T cell responses (154–156). Furthermore, immunosuppressive, TGF-β1-producing, CD4^+^ type 3 helper T (T_h3_) cells as well as CD8^+^ T_reg_ cells and IL-10 producing regulatory B (B_reg_) cells have also been documented (146).

T_reg_ cells have been considered as living drugs for the treatment of autoimmune and inflammatory diseases (146) as they have been used to reconstitute patients with AID after *ex vivo* expansion. To this end, T_reg_ cells need to show consistent immunosuppression potential, prolonged *in vivo* survival and stability of the phenotype (146). Nevertheless, T_reg_ cells have shown phenotypic plasticity, potentially related to the diverging contextual conditions they encounter in different tissues (e.g., cytokine milieu, availability of antigen, TcR affinity etc.). The level of immunoregulation likely will need to be customized for each AID and inflammatory disorder (146), and to be balanced against unwanted, non-specific immunosuppression, that would increase the risk for opportunistic infections or malignancies. Because the CD25^+^ FOXP3^+^ T_reg_ cells are very amenable to *in vitro* expansion, they have been used in several clinical trials, including treatment of T1D patients (146, 157–159). In some cases, the infused T_reg_ cells remained stable and detectable for 1 year. In other cases, the effects were rather transient and did not provide a therapeutic benefit (146). One possible explanation for this limited success could be related to the fact that the large numbers of transferred cells were not selected on the basis of any antigen-specificity and therefore, they probably harbored only very small amount of T_reg_ cells specific for the antigens involved in the autoimmune process.

FOXP3^+^ T_reg_ cells have constitutive expression of CD25 in marked contrast to conventional T cells that only express this protein following activation. This difference has prompted the search for therapeutic products based on modified and/or low dose IL-2. The rationale behind this is that a low dose of IL-2, especially when the cytokine is modified to increase its binding to CD25 and diminish that to CD122 (β chain of the IL-2 receptor) will engage IL-2 receptors (expressing the α/β/γ IL-2 receptor chains) on T_reg_ cells and promote their expansion without activating T and NK cells (expressing β/γ IL-2 receptor chains) (160). Indeed, low-dose IL-2 has shown a satisfactory safety profile in human and is currently being tested in several AID (160). To increase the circulating half-life of these products, the muteins are generally engrafted into an irrelevant mAb or fused to an immunoglobulin Fc portion (160). Alternatively, some investigators have proposed the use of complexes of IL-2/anti-IL-2 mAb that also increase the half-life of circulating IL-2 and release the cytokine in the proximity of high affinity α/β/γ IL-2 receptors (161).

It is expected that the *in vivo* expanded polyclonal T_reg_ cell populations will contain a sizable amount of autoantigen-specific T_reg_ cells, as consequence of the ongoing chronic response, but these therapeutic principles are not designed to favor any antigen specificity and thus, the same risks of inducing immunosuppression that were associated to the polyclonal T_reg_ cell transfer may also apply for this situation. Thus, finding approaches to promote an optimal balance between disease- and pathogen-specific T_reg_ cell activation continues to be a critical challenge for the clinical development of this cytokine therapy (160). A potential solution to avoid broad immunosuppression could be a localized delivery of IL-2. https://pandiontx.com is currently using such strategy by fusing an IL-2 mutein to the Fc chains of a mAbs directed to MadCAM. This engineered biologic is expected to focus IL-2 on endothelial surfaces of the gastrointestinal tract and hence, expand locally T_reg_ cells and provide a benefit for patients with IBD. Furthermore, a recent investigation has shown that chemical inhibition of the cyclin-dependent kinase 8 (CDK8) and CDK19, or knockdown/knockout of the *CDK8* or *CDK19* gene, can induce FOXP3^+^ T_reg_ cells in mice (162). The conversion which resulted from the release of FOXP3 repression, was TGF-β1-independent, not affected by inflammatory cytokines and provides a new opportunity to expand T_reg_ cells *in vivo* (162).

Polyclonal and antigen specific T_r1_ cells have been also used in clinical trials (163–166). However, as the CD25^+^ counterparts, they have shown limited survival capacity *in vivo* (164). This limitation has prompted alternative designs based on the generation of conventional antigen-specific CD4^+^ T cells that are converted into T_r1_ cells by lentiviral transduction of *IL10*, and an additional gene, which could be included in a bidirectional vector to provide a convenient marker for purification (167, 168). Alternatively, https://www.sangamo.com is trying to solve the issue of antigen selectivity by developing regulatory-chimeric antigen receptor T (CAR-T_reg_) cells (169–172). Their original target indication is transplant rejection and thus, they aim to generate CAR-T cells using donor-human leukocyte antigens (-HLA) binding elements (i.e., variable fragments of anti-HLA mAbs) engineered in a chimeric molecule that express intracellular domains of CD28 and CD3 molecules, which are transfected into highly purified T_reg_ cells isolated from the host patient (173–176).

## The Promise of Immune Tolerance

Despite the recent advances in the immunotherapy field, there is still a significant correlation between the increased efficacy of these novel medicines with the undesired risk of infections. This relationship is understandable, since none of the existing approaches is exclusively directed to the specific triggers of the disease. At most, immunotherapy principles target cell subsets or soluble factors that are predominantly involved in the autoimmune attack, but nevertheless are also needed to fight pathogen invasion. Thus, the necessity to develop truly transformative therapies, that specifically control and provide curative potential for AID, without broad immunosuppression remains actual, and is prompting a resurge of the long-sought immune tolerance field.

Induction of immune tolerance to self-antigens occurs naturally in the thymus and bone marrow during T and B cell ontogeny (177, 178). These processes are not completely efficacious, since they permit the development and subsequent export to periphery of lymphocytes with low avidity for autoantigens (179). Escaping autoreactive clonotypes are normally silent in the body (180, 181), but they can be awakened when their thresholds of activation are reduced; i.e., due to cross-reactivity with pathogen or commensal organisms in the context of immunological danger that may be favored by the host expression of AID-predisposing genetic variants (182). Specific intervention in autoantigen-specific immune function, to enhance or restore the mechanisms of immune tolerance, represents an excellent opportunity to fulfill the huge medical need existing in AID therapy (183). Such intervention could be, in principle, attempted targeting either the central or peripheral sites where immune tolerance is orchestrated.

### Central Tolerance

Induction of central tolerance, i.e., by resetting the immune system, is a possible option, although associated with very harsh procedures (184, 185). This notion developed following reports of patients that underwent hematopoietic stem cell transplantation (HSCT) to treat hematological malignancies and showed remissions of coincident AID (186, 187). HSCT following extensive immune ablation restores the immune system and provides long-term remission rates in some AID patients (188). However, the risk-benefit ratio of these therapies has to be carefully and individually evaluated, since the procedures expose the patients to temporary iatrogenic severe immune deficiency and are, overall, associated with unacceptable mortality (188, 189).

### Peripheral Tolerance

Immune tolerance approaches directed to the peripheral immune system seem to be more amenable from the safety and practical point of view. However, these strategies are still in preclinical phases of development or have not yet demonstrated conclusive long-term efficacy in the clinic. The landscape of peripheral immune tolerance approaches has been recently reviewed (190), including some points of view on how to maximize their development and smooth progression to the clinic (183). Thus, this section will only comment on the different mechanisms of action of a few representative therapies without entering into the details of the specific products.

Therapeutic principles to induce peripheral immune tolerance have been historically grouped into (i) induction of clonal deletion and/or anergy or (ii) promotion of active regulatory mechanisms.

Deletional approaches might work well in situations where there is a limited immunogenic repertoire, as it the case of immune responses to immunotherapy products (191). However, these will face major challenges in the context of AID, which are driven by a complex autoimmune repertoire that is almost impossible to completely determine. The rationale for deletion approaches was triggered by a concept of hierarchy in the development of autoimmune responses, which postulates the existence of major immunodominant epitopes that initiate the immune response and precede broad epitope spreading (192–195). Eliminating or inactivating the T cell clones directed to these primary disease-related epitopes was suggested to suffice in aborting further disease progression and stopping autoimmunity in mice (196, 197). Indeed, there is evidence that reactivity to certain antigens, i.e., insulin or glutamic acid decarboxylase (GAD) in T1D, is often observed before the autoimmune response expand to other autoantigens (198). However, it would be too risky to assume that eliminating a very restricted antigen repertoire will be sufficient to halt the entire complex autoimmune response. This could be particularly difficult in the human population, where the MHC, although strongly associated to certain AID, is still very diverse and thus, allows presentation of multiple autoantigen epitopes in the context of different binding registers (197) that might even derive from post-translationally modified or hybrid peptides (199). It is also reasonable to expect that less immunodominant epitopes could drive AID once the stronger reactivities would be eliminated, provided this could be achieved completely.

**Figure 3:**
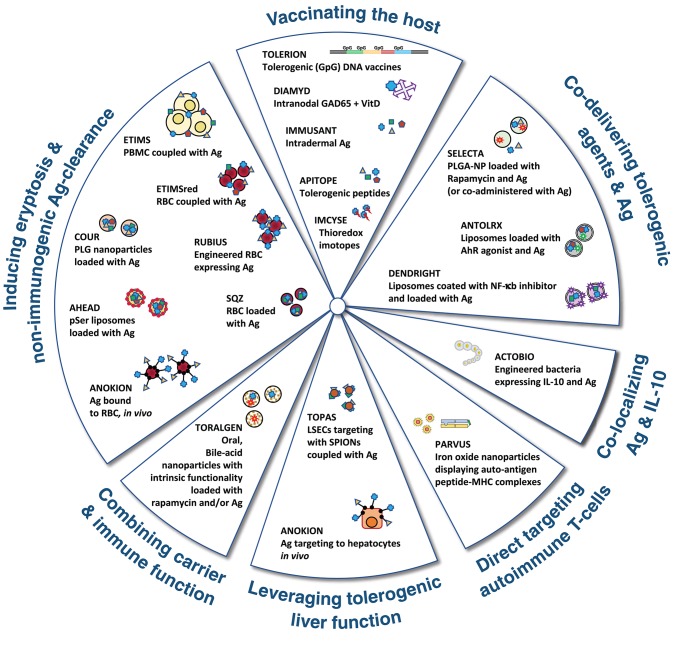
Graphical representation of the major approaches being followed by pharma and biotech companies to induce immune tolerance. Antigen (Ag) could be one or more whole proteins or derived peptides involved in the autoimmune response. Further explanation on the different MoA is presented in the text.

The alternative options to promote regulatory responses have classically selected one or a few discrete autoantigen epitopes. The choice has been determined by the specific indication (i.e., aiming to induce tolerance to therapeutic substances with high immunogenic potential) or by the conviction that truly regulatory responses directed to a single/discrete epitope/s involved in a given autoimmune disorder, are necessary and sufficient to control all disease causing reactivities in a bystander manner (183, 200). Therapeutics based on bystander regulation are intrinsically more feasible since they only require the knowledge of some disease associated autoantigens, which is available for most of the AID. On the other hand, it has to be noted that strictly classifying immune tolerance approaches into deletion or regulatory type is rather artificial. Often such choice is guided by the original scientific concept that prompted the therapeutic intervention. However, *in vivo*, these mechanisms probably overlap to a certain extent and immune tolerance might result from the concerted action of several different MoA (201, 202). A schematic representation of different approaches used to induce peripheral immune tolerance is depicted in Figure 3.

#### Inducing Eryptosis and Non-immunogenic Antigen-Clearance

Induction of tolerogenic responses that deleted clonotypic T cells were induced by coupling specific antigens to syngeneic lymphoid cells already four decades ago (203). Currently, the believe is that tolerogenic responses mediated by peptide antigens coupled to cells do not rely exclusively on induction of anergy or clonal deletion but on the parallel induction and expansion of regulatory T cells, which can also exert suppression, in the context of the affected organ, in a bystander manner (201, 204). These concepts have inspired a series of therapeutic approaches aiming to promote immune tolerance in AID patients. For example, the group led by Roland Martin infused MS patients with autologous PBMC, which were previously coupled with a cocktail of seven myelin peptides (Establish Tolerance in MS; ETIMS) (205). The same group, http://nims-zh.ch/etimsred.html, has initiated a subsequent clinical trial (ETIMSred) replacing PBMC by autologous red blood cells (RBC) (206). Both trials demonstrated feasibility of the approach and were safe and well-tolerated. Furthermore, *ex vivo* studies using PBMC from treated patients showed some reductions in recall T cell responses to myelin peptides (205, 206). Another scientific group has used a sortase-A mediated reaction to covalently link autoantigen peptides to mouse RBC and to, following re-infusion of these modified RBC, demonstrate protection toward experimental autoimmune encephalomyelitis (EAE) and T1D disease (207).

Several biotechnology companies are currently developing additional therapeutic products that use RBC as a carrier for autoantigen delivery: https://www.rubiustx.com is developing engineered RBC from autologous hematopoietic precursor cells that express AID-related antigens on their surfaces, and http://sqzbiotech.com is loading antigens into RBC by temporary disrupting the membrane of these cells, during a forced passage through microfluidic devices that contain antigen cocktails (208). These approaches are based on the principle of eryptosis (209), which ensures an orderly elimination of senescent or damaged RBC that is accompanied of regulatory instructions supporting the induction of anergic and/or tolerogenic responses (210). In this line, and taking advantage of the scrambling processes occurring in the cell membranes during apoptosis (211), scientists at http://www.aheadtherapeutics.com are developing a nanotechnology based on phosphatidylserine liposomes. These liposomes that additionally encapsulate autoantigens, are taken up by APC as if they would be apoptotic cells and thus, the cargo is presented to T cells in a tolerogenic manner (212, 213). A further strategy, built on existing physiologic mechanisms for clearing apoptotic debris, is pursued at https://www.courpharma.com. In this case the approach is based on proprietary biodegradable poly[lactide-co-glycolide] (PLG) microparticles (circa 500-nm diameter) that encapsulate autoantigens and are functionalized on their surfaces to enhance their uptake by the mononuclear phagocyte system (214). It seems that the uptake of these microparticles for subsequent cargo processing and presentation to T cells in a tolerogenic manner (215, 216) is mediated by the scavenger receptor MARCO (217), which is abundant on the surface of marginal zone macrophages (218).

All the technologies indicated above require an *in vitro* or *ex vivo* loading of the antigenic peptide into/on the carrier cells or particles before transferring (generally by i.v. injection) the drug substance to patients with AID. An alternative to this laborious process is being considered at https://anokion.com. Their approach consists on the direct, *in vivo*, targeting of autoantigens to RBC. This intervention is accomplished by conjugating the selected proteins to a synthetic peptide (ERY1) that is specific, and displays high affinity, for glycophorin-A (GYPA), which is a surface molecule exclusively expressed on erythrocyte surfaces (219). In the case of small peptide antigens, the autoantigen focusing to RBC is accomplished by fusing these peptides to a single-chain Fv (scFv) antibody fragment (TER119) that is also directed to GYPA (219). Initially, the administration of these GYPA-targeting biologics was reported to induce tolerance to the linked antigens in mice, via deletion and/or anergy of cognate T cells (191, 219). More recently, these authors have also shown preclinical evidence for T_reg_ cell induction using the same principles (220).

#### Vaccinating the Host

Development of immune tolerance has also been attempted by direct administration of the antigen, both in the form of proteins or peptides or by using DNA vectors encoding these proteins. DNA vaccination is the approach selected at https://tolerion.bio. The company is developing DNA vaccines for intramuscular delivery using plasmids encoding different autoantigen epitopes that have the immunostimulatory CpG motifs replaced by immunoinhibitory GpG (221, 222).

Several approaches, mostly initiated by academic groups, aiming to induce immune tolerance using whole protein autoantigens or derived peptides have reached the clinic and although the approaches were shown to be safe, their insufficient efficacy did not support progression beyond phase 1 or 2 trials. These were recently reviewed by Serra and Santamaria elsewhere (190) and thus here only a few examples of biotech companies that remain actively engaged in clinical trials are discussed. https://www.diamyd.com is among the pioneers in treating T1D patients with a disease autoantigen. The initial intervention which included pediatric/juvenile patients consisted of multiple daily s.c. injections of glutamic acid decarboxylase 65-kilodalton isoform protein formulated in alum adjuvant (GAD-Alum). The treatment was well-tolerated but did not meet the overall expectations (223, 224). The group has continued with their immune tolerance program combining the original product GAD-Alum with immunomodulators such as Vit D and/or changing the route of administration (i.e., intranodal injections). https://apitope.com has based their immune tolerant products on the selection of peptides (apitopes) that can elicit T cell responses without any further processing by the APC (225). The group has shown encouraging results in MS (226) and is pursuing additional AID indications. Another approach, using cocktails of immunogenic peptides delivered intradermally in the absence of adjuvants has been developed at http://www.immusant.com. The main focus of the company was on celiac disease (CelD) and the team delivered an encouraging phase 1 trial (227), which was safe and well-tolerated. However, the company has announced recently the discontinuation of the ongoing phase 2 study after reviewing the results of an interim analysis.

In most cases, the therapeutic intervention for the protein and epitope-based therapies involves the production of IL-10 and often, this signature is also associated to increased frequencies of T_reg_ cells. In some cases, the vaccination has been proposed to induce a switch from inflammatory T_h1_ profile toward anti-inflammatory types. In other cases, it is postulated that the antigen presentation of the immunogenic epitopes is performed by immature APC that deliver tolerogenic instructions to the cognate T cells and skew them into T_reg_ cells. Additional MoA can be induced using vaccination approaches. For example, http://imcyse.com is aiming to induce cytotoxic/cytolytic T cell responses to APC displaying disease related pMHC on their surfaces, *in vivo*. The therapy is based on s.c. administration of modified autoantigen peptides that are flanked by thioredoxin-like motives to augment pMHC-TcR interactions and promote the differentiation of cognate CD4^+^ T cells into cytolytic T cells. Specific killing of pMHC-loaded APC by the expanded “cytolytic killers” also induces the death of other disease-related T cells engaged with the same APC. Furthermore, such action prevents the activation of any disease-specific T cell by active removal of the APC source (228). A phase 1 trial in recent onset of T1D patients has been reported by the company with no safety concerns.

#### Co-delivering Tolerogenic Agents and Antigen

Delivering autoantigens alone is probably the simpler way to induce tolerance. However, such method rely considerable on the selection of the right peptide and potential adjuvant, as well as on the dose, frequency and route of administration, since ultimately the immune system of the treated patients, that is poised to promote effector responses, would need to interpret this new source of antigen and mount a regulatory response. This difficulty to predict the *in vivo* fate of the immunogenic proteins has triggered further therapies that deliver the autoantigens together with specific instructions to promote regulatory responses. One of those include the loading of the immunogenic cargo into autologous “tolerogenic” APC. Frequently, dendritic cells (DC) are differentiated from peripheral blood monocytes in the presence of modulatory agents that skew these cells toward anti-inflammatory/tolerogenic phenotypes or that remove some costimulatory elements before reinfusion into patients (190). Alternatively, and to avoid laborious cell therapy processes, the autoantigens could be delivered using nanoparticles that simultaneously incorporate compounds known to drive T_reg_ cell differentiation. Among others, rapamycin (229), aryl hydrocarbon receptor (AhR) agonists (230–232), and inhibitors of nuclear factor kappa-light-chain-enhancer of activated B cells (NF-κB) (233, 234) have been described to induce tolerogenic phenotypes on DC.

https://www.selectabio.com is developing a platform based on polylactic acid (PLA) and poly(lactic-co-glycolic acid) (PLGA) nanoparticles that incorporate rapamycin and autoantigen, or only rapamycin if the autoantigen is co-injected (s.c.) with the tolerogenic nanoparticle (235–239). The company has successfully completed phase 2 trials to induce tolerance to exogenous administered uricase in patients with gout and is advancing in other indications. https://antolrx.com has chosen to deliver the AhR ligand, 2-(1′H-indole-3′-carbonyl)-thiazole- 4-carboxylic acid methyl ester (ITE), together with autoantigen. Initially the two components were intended to be co-delivered by simultaneous coupling to the surface of gold nanoparticles (240, 241). Following a collaboration with https://www.pfizer.com the team has switched to liposomes as the preferred carrier vehicle (242). https://www.dendright.com.au on the other hand, utilizes a platform that incorporates NF-κB inhibitors in the lipid bilayer of liposomes and autoantigens in the hydrophilic core (243). The company has completed recently a phase 1 trial in RA patients using DEN-181; a liposome product containing calcitriol and a collagen II peptide (244).

#### Co-localizing Antigen and IL-10

In addition of delivering antigens, nanomedicine approaches have also aimed to deliver anti-inflammatory cytokines to reverse ongoing immune responses. For example, TGF-β1 and IL-2 have been encapsulated into PLGA nanoparticles to promote T_reg_ cell responses in mice (245). On the other hand, based on many reports using human and mouse cells *in vitro* as well as mouse models of disease, IL-10 has emerged as a key cytokine for dampening autoimmune responses. Such knowledge prompted its use in clinical trials for Crohn's Disease. Unfortunately, systemic IL-10 treatment to AID patients was not effective and rather resulted in considerable side effects (246, 247). These results encouraged the consideration to target IL-10 delivery to the inflammatory site minimizing systemic exposure. Scientists have explored different ways to locally deliver IL-10. For example, different densities of DEC-205 ligands for targeting, ovalbumin-encapsulating, nanoparticles to DC have been used to regulate IL-10 production by these cells (248). Alternative, other authors have encapsulated plasmids encoding IL-10 into poly[epsilon-caprolactone] (PCL) large (200 nm) nanoparticles for oral delivery and treatment of IBD in mice. These plasmids were released in the intestinal tract, endocytosed by cells of the intestinal lumen and subsequently, transcribed for local IL-10 expression (249). Gut expression of IL-10 is also the strategy followed by https://actobio.com, a biotechnology company that has opted for the use of genetically modified bacteria instead of nanoparticles. Particularly, they have replaced the thymidylate synthase gene of *Lactococcus lactis* by a synthetic sequence encoding human IL-10 (250). This company has successfully tested the safety of the approach, including biologic containment in a phase 1 clinical trial (251). The same group is also developing other products based on *L. lactis*, encoding nanobodies to human TNF-α (252) and is investing on immune tolerance programs for CelD (253) and T1D (254–256), by providing simultaneous bacterial expression of the autoantigen and the anti-inflammatory cytokine. In the case of TID, best preclinical data was always observed when the oral treatment with the engineered bacteria was complemented with systemic (i.v.) treatment with anti-CD3 mAb (254–256). Such preclinical evidence, together with the recent report on human T1D therapy using Teplizumab (39), suggests that a combination of these two agents would be a good approach to intervene in human T1D.

#### Direct Targeting of Autoimmune T Cells

A completely different strategy for the induction of immune tolerance is followed by https://parvustx.com. The platform of this biotech company is based on the development of Navacims, consisting of iron oxide nanoparticles that display large density arrays of pMHC. These pMHC are selected on the basis of known disease-related T cell epitopes that associate with MHC class II molecules displayed by patients suffering from AID. The Navacims engage directly microclusters of TcR on cognate, antigen experienced, CD4^+^ T cells in the absence of any costimulatory signal and instruct these cells to become T_r1_ cells (257, 258). These T_r1_ cells expand *in vivo* and are subsequently activated exclusively in the attacked tissue and its draining lymph nodes, where they locally suppress all disease-causing T_eff_ cell responses with no systemic effects. Efficacy of Navacims is mediated by IL-10, IL-21, and TGF-β1 and also involves functions of B_reg_ cells. Navacims have been shown to prevent disease progression in pre-diabetic non-obese diabetic (NOD) mice and reverse disease in already diabetic NOD mice (257). Mice treated with Navacims remain capable of mounting immune responses to pathogens and to develop antibody responses to disease unrelated antigens following vaccination (257). Human-specific Navacims expand human T_reg_ cells in NOD- *Scid*-common gamma chain deficient (NSG) mice reconstituted with PBMC from T1D patients (257). Furthermore, the induction of Navacim-specific T_reg_ cells provides a biomarker (using tetramers made of the same pMHC included in the Navacim) to guide human dosing and to decide timing of repeat treatment course (“booster” regimen) for maintenance of immune tolerance. A Navacim project for T1D is being developed in collaboration with https://www.novartis.com. Additional Navacim tool compounds have shown efficacy in mouse models of MS, RA, primary biliary cholangitis (PBC), primary sclerosing cholangitis (PSC) and autoimmune hepatitis (AIH) (257, 259). Navacims to treat CelD, IBD, and autoimmune liver diseases are pursued together with https://www.gene.com. Navacims display exquisite selectivity for the organ and disease being targeted. However, to ensure a large patient coverage, it would be required to develop more than one product per disease. This could be easily accomplished in situations like T1D where disease is tightly associated to certain HLA DRB1 loci. Conditions with more diverse DRB1 associations might require the use of oligomorphic HLA loci, such as DRB3, DRB4, or DRB5 (259).

#### Leveraging Tolerogenic Liver Function

A feature of most types of particles, that is also shared by apoptotic cells, is their accumulation in the liver. The liver is the largest solid organ of the human body and it is generally regarded as a fundamental metabolic organ. However, the liver is also a core component of the immune system that has key relevance removing pathogens and exogenous antigens from the systemic circulation and promoting tolerance toward these agents (260–262). The liver is strategically positioned at a crossroad between the intestine and systemic circulation. It receives blood supply from the hepatic artery containing naïve and memory lymphocytes and from the portal vein, which is rich in food and microbial antigens. These gut-derived constituents are efficiently cleared by cells of the hepatic reticuloendothelial system such as fenestrated liver sinusoidal endothelial cells (LSECs), Kupffer cells, and DC, which also present them to passenger T cells in a tolerogenic context. Hepatic APC secrete abundant TGF-β1 and IL-10, express PD-L1 and contribute to the differentiation of T_reg_ cells (260–262). Thus, the liver immune system favors induction and maintenance of tolerance over immunity, avoiding immune responses to harmless foreign antigens form the diet and this function is believed to be largely mediated by LSECs (263–268). Based on these observations, https://topas-therapeutics.com is developing a nanomedicine platform aiming to focus autoantigens to LSECs. This company generates super paramagnetic iron oxide nanoparticles (SPION) of about 10 nm diameter that are conjugated with up to 100 autoantigen peptides (269). The small size of these particles is a feature for LSEC targeting. LSECs display poor phagocytic capabilities and preferentially take up soluble macromolecules and small particles (270). Electron microscopy of liver sections from mice treated with SPIONs have been shown how these nanoparticles localize into the endosomal compartment of LSECs (269). Treatment with SPIONs coated with myelin basic protein (MBP) or myelin oligodendrocyte glycoprotein (MOG) peptide protected mice from EAE and improved disease scores in mice with established disease (269). Interestingly, disease protection was also achieved in splenectomized mice, indicating that the spleen was not required for the induction of the regulatory response although it played a role in maintenance of tolerance (269). The relevance of hepatic APC promoting immune tolerance has triggered a new research line at https://anokion.com. Adding to the erythrocyte binding technology described above, further binding principles based on N-acetyl galactosamine (GalNAc) or N-acetylglucosamine (GluNAc) that target receptors expressed by hepatic APC are now described (271). Binding and internalization of galactose (Gal) or N-acetyl galactosamine (GalNAc)-terminating glycoproteins by hepatocytes is often mediated by the C-type lectin receptor, asialo-glycoprotein receptor (ASGPR) (272), which is a feature often used in gene therapy approach to deliver the therapeutic principles to hepatic cells (272). Interestingly, scientists at the Baylor Institute for Immunology Research have demonstrated that focusing of antigens to DC using antibodies directed to a type of ASGPR expressed on these cells is a very effective method to generate antigen-specific IL-10-producing T cells with suppressive capabilities (273).

#### Combining Carrier and Immune Function

The liver is intimately linked to the gut and overall intestinal tract and the composition of the microbiome represents another node of crucial relevance preventing or inducing autoimmunity (274). The gut microbiota can control gut inflammation by preventing or promoting T_reg_ cell responses (275). There are also reports demonstrating that, based on molecular mimicry, CD8^+^ T cell responses against microbial antigens that protect mouse and human hosts against colitogenic insults are also linked to T1D (276). It is conceivable to think that many more cases of cross-reactivity between human and microbiota antigens exist, and that evolutionary regulatory responses that prevent exacerbated responses to commensal bacteria also support tolerance to self-antigens. Conversely, mechanisms promoting the elimination of certain microbiota could result in autoimmunity in patients with a predisposing HLA. Furthermore, metabolites produced in the gut by the host or microbiome may also play an important role controlling homeostasis at distant sites. A good example of metabolic interaction between host and microbiome is provided by the production of bile acids (BA), which are initially produced as primary BA from cholesterol in the liver and subsequently, modified into secondary BA in the intestine by commensal bacterial (277). Both types of BA are capable on interacting with, farnesoid-X-receptor (FXR) and G protein-coupled bile acid receptor 1 (GPBAR1), although the preferences of primary BA are for FXR and secondary BA prefer GPBAR1 (277). Because the function of different BA receptors could be immune counterregulatory the balance of the different BA species due to alterations in microbiota composition or following exogenous administration or one or other type of agonist could be very important maintaining immune homeostasis (278). In this regard, the platform announced by https://toralgen.com that proposes the delivery of multiple types of cargo using oral nanoparticles made of polymerized ursodeoxycholic acid (pUDCA) (279), represents an advance toward the use of vehicle nanocarriers with intrinsic therapeutic function.

## Safety Aspects

In this review, we have discussed three major types of therapies aiming to ameliorate AID, namely biologic immunotherapy, cell (and gene) therapy, and immune tolerance. All of these emerged with the intention to close the therapeutic gap resulting from the limited availability of medicines capable of delivering a substantial clinical benefit in the absence of adverse effects (280). Biologics were pioneers in this attempt and represent today a major part of the established drugs used for autoinflammatory and AID. These drugs have an exquisite selectivity for their targets, which is a major difference over the classical low molecular weight remedies. Nevertheless, they target immunocompetent cell subsets, soluble factors, or pathways, which are essential in host resistance to pathogens, and therefore they also carry the risk of increasing the susceptibility to opportunistic infections. This problem turned out to be a major concern for the development of the first TNF-α blockers (281), and it is still today a significant cause of morbidity for many immunotherapies (9, 10, 282, 283). Moreover, in the context of obtaining an adequate immunological balance, TNF-α blockers intended for the treatment of RA have elicited SLE (284–286). Over the years, the scientific community has learned that immunotherapy products do not represent universal solutions for all patient populations and that their indiscriminate use could be detrimental for some of them. Different drugs have shown to be efficacious in some diseases, but of limited benefit in others, which is in line with the recent views classifying autoinflammatory and autoimmune disorders according to hierarchic cytokine pathway maps (7, 11). Furthermore, the existence of multiple endotypes within a given disease is recognized (287, 288), and thus, there is a need to accurately diagnose the appropriate disease subtype and provide the right treatment to the right patient at the right time (289).

In addition to the specific target effects, therapeutic proteins, particularly when they contain non-human sequences and/or are repeatedly administered, can be immunogenic and promote host anti-drug antibody (ADA) responses. The clinical utility of immunotherapeutics may be impacted by these ADA, since they could neutralize the therapeutic effects of the medicine and/or induce hypersensitivity reactions in the treated patient (290, 291). Strategies to mitigate these risks included switching to a different molecule with similar targeting capabilities, engineering the product to eliminate as much as possible the non-human sequences (i.e., mouse antibody framework), reducing the chance to generate protein aggregates, eliminating Fc receptor binding ability or inducing immune tolerance. Most of these solutions are already incorporated during the design of the new drugs, however when these predictions fail and ADA responses occur, the removal of ADAs could be a real challenge and could result in a discontinuation of the product.

As discussed in this review, cell therapies intended to induce regulatory responses could be polyclonal (antigen agnostic) or antigen-specific. Polyclonal T_reg_ cells will be able to efficiently suppress T_eff_ cells of various specificities (bystander suppression), however they will also indiscriminately affect the host defense response (dominant suppression) and therefore, the same risks of infections related to some of the biologics will apply (145, 146). Antigen-specific cell therapies will be safer. However, if they result from genetic engineering of the regulatory cell populations other risks associated to uncontrolled expansion and extensive bystander suppression may develop. One extreme of non-specific immune dysregulation, could be severe acute immunosuppressive effects mediated by cytokine release syndrome of CAR-T_reg_ cells, resembling the immunostimulatory outcomes observed with the complementary effector CAR-T cell therapies in oncology (292). Furthermore, T_reg_ cells may still display a considerable phenotypic plasticity and consequently, there is a risk that the inflammatory conditions ongoing in the treated patients (e.g., cytokine milieu, availability of antigen, concomitant therapy) affect the phenotype of the transferred cells, reverting them into subsets with potential to exacerbate disease. Thus, the level of required immunosuppression likely will need to be customized for each autoimmune and autoinflammatory disorder and to be balanced against unwanted, non-specific immunosuppression (146).

Some of the approaches currently followed to induce immune tolerance were inspired by the antigen desensitization protocols established for the treatment of allergy. These therapies aimed originally to switch the ratios of allergen-specific IgE toward the non-anaphylactogenic IgG4 class, rather than searching for a true induction of tolerance that should have dampened all antibody responses (293). To achieve such goal, atopic patients were exposed, over long periods (months to years), to gradually increasing doses of allergen. Such process implied the administration of the whole allergen which represented a high risk of anaphylaxis. In general, autoimmune responses are not associated to IgE, and the administration of autoantigen protein or peptides used in the immune tolerance protocols does not favor the induction of autoantigen-specific IgE reactions. However, IgG-mediated anaphylactic responses could develop upon repeated exposure of the antigen (294) as detected for some therapeutic antibodies (295) and thus, these could also evolve during antigen-specific tolerance protocols that require repeated administration of proteins or peptides alone or attached to the surfaces of nanoparticles. Furthermore, therapies that expose patients to the same immunogenic agents involved in the autoimmune attack have the inherent risk of provoking disease exacerbation rather than protection. The challenge is particularly high since these safety concerns may not be optimally extrapolated from preclinical animal studies, even from non-human primates, because both clinical intervention and potential disease exacerbation are strongly linked to pre-disposing MHC alleles/haplotypes that are specific to human. Therefore, careful design for first in human, phase 1 trials, is essential and it should pay increased attention to the selection of dose and frequency of administration. Moreover, the status of the individuals in phase 1 studies is also critical, since choosing healthy individuals displaying HLA alleles/haplotypes not associated with the AID might be irrelevant to obtain any preliminary indication of target engagement and/or efficacy for the drug candidates under evaluation. On the other hand, choosing healthy individuals expressing HLA alleles/haplotypes related to the disease might expose them to un-ethical drug exposure that might trigger unwanted immune responses. Such argumentation would support the selection of patients from the very onset of clinical testing. However, an acceptable approach would be to opt for patients with only subclinical disease or for patients with long-lasting disease. Both situations offer advantages and inconveniences for clinical evaluation and the choice will be determined by the MoA of the test drug and specific trial design. These studies would benefit decisively of having exploratory and clinically accepted biomarkers that can predict the outcome of the trial. In addition, a cautious clinical approach that takes into account the specific MoA of each therapy will be required to advance the different programs.

## Conclusion

This review has discussed some of the existing and developing immunotherapy and precision medicine approaches aiming to ameliorate and/or cure AID. The established immunotherapies for AID are the less specific options whereas highly sophisticated antigen-specific alternatives are still under development. Immunotherapy is particularly useful for autoinflammatory conditions, where the antigenic trigger is still elusive, or the disease is mediated by multifactorial activation of several inflammatory pathways like those found in cryopyrin-associated periodic syndrome. These therapies are also very efficient in some selected AID, like Pso where they can deliver extraordinary efficacy results with almost absence of adverse effects. Unfortunately, immunotherapies for all AID do not exist and, when they are available, they still rely heavily on immunosuppression that is associated with increased susceptibility to opportunistic infections. Cell therapies represent a bridge between immunotherapy and immune tolerance since these living drugs have the potential to adapt to the host environment and produce a repertoire of factors and immune functions of benefit for the affected patient. However, in most cases they are not specific and/or involve logistically complicated development processes that are patient-personalized. The emerging immune tolerance alternatives do have the potential to address all the missing aspects of the former therapies and to provide a truly transformative and game changing option for the treatment of AID.

## Future Perspectives

It is expected that immunotherapies for AID will continue to evolve. They will (i) incorporate new target specificities, i.e., immune checkpoints, (ii) increase in sophistications to enhance efficacy and reduce adverse effects, i.e., utilizing bispecific molecules and selectively using them in optimal disease endotypes, and (iii) gain acceptance as first line treatment option, i.e., with increased use of biosimilars. Immunotherapeutics are expected to be the next current standard of care and to significantly raise the requirements for clinical improvement of newly developed medicines. Cell-based personalized therapies might find their niche in the near future for the treatment of AID. However, their development processes will require substantial improvements to accelerate production, combine efficacy features, incorporate safety switches and reduce costs to ensure broad availability to all patients in need. The current assortment of immune tolerance approaches makes it difficult to predict which of them will provide the most effective MoA. Hence, it is anticipated that one or several of these strategies will succeed in delivering the long-awaited curative solution. “…rien n'est plus imminent que l'impossible, et que ce qu'il faut toujours prévoir, c'est l'imprévu" (296).

## Author Contributions

JC, CRe, CRa, LR, DP, and MK contributed to the writing of the manuscript, are accountable for its content, and approved its publication.

### Conflict of Interest

All authors are employees of Novartis and are engaged in drug development and/or translational medicine activities aiming to develop new therapeutics for AID. Novartis has a research and development collaboration with Parvus Therapeutics in the field of T1D.

## Footnotes

^1^https://www.amgen.com

^2^https://www.astrazeneca.com/
